# Evaluation of an electronic consultation service in psychiatry for primary care providers

**DOI:** 10.1186/s12888-018-1701-3

**Published:** 2018-05-02

**Authors:** Douglas Archibald, Julia Stratton, Clare Liddy, Rachel E. Grant, Douglas Green, Erin J. Keely

**Affiliations:** 1 Department of Family Medicine, University of Ottawa, Ottawa, ON Canada; 2 Bruyère Research Institute, 85 Primrose Avenue, Ottawa, ON K1R 6M1 Canada; 3 Department of Psychiatry, University of Ottawa, Ottawa, ON Canada; 4 Faculty of Education, University of Ottawa, Ottawa, ON Canada; 5 Department of Medicine, University of Ottawa, Ottawa, ON Canada; 6 Ottawa Hospital Research Institute, Ottawa, ON Canada

**Keywords:** Electronic consultation, eConsult, Primary health care, Psychiatry

## Abstract

**Background:**

This study explores the effectiveness of an electronic consultation (eConsult) service between primary care providers and psychiatry, and the types and content of the clinical questions that were asked.

**Methods:**

This is a retrospective eConsult review study. All eConsults directed to Psychiatry from July 2011 to January 2015 by Primary care providers were reviewed. Response time and the amount of time reported by the specialist to answer each eConsult was analyzed. Each eConsult was also categorized by clinical topic and question type in predetermined categories. Mandatory post-eConsult surveys for primary care providers were analyzed to determine the number of traditional consults avoided and to gain insight into the perceived value of eConsults.

**Results:**

Of the 5597 eConsults, 169 psychiatry eConsults were completed during the study period. The average response time for a specialist to a primary care provider was 2.3 days. Eighty-seven percent of clinical responses were completed by the psychiatrist in less than 15 min. The primary care providers most commonly asked clinical questions were about depressive and anxiety disorders. 88.7% of PCPs rated the eConsult service a 5 (excellent value) or 4.

**Conclusions:**

This study indicates that an eConsult psychiatry service has tremendous potential to improve access to psychiatric advice and expand the capacity to treat mental illness in primary care. Future research may include follow-up with PCPs regarding the implementation of specialist advice.

## Background

Wait times to access mental healthcare services remains a concern in Canada, where the average wait time for psychiatric service is 20.8 weeks [1]. Wait times vary drastically between provinces, with the shortest wait times being in British Columbia (16.3 weeks) and the longest in Newfoundland & Labrador (83.0 weeks). Canadians living in rural and remote communities also face a unique set of challenges. The Canadian Mental Health Association suggests the rates of Major Depressive Disorder (MDD) are higher amongst those living within rural and remote regions, yet these individuals face a number of barriers to receiving psychiatric care within their own communities, such as geographical barriers, limited health resources and services, and challenges in recruiting and retaining healthcare professionals [2].

In an effort to improve access to psychiatric care, curbside consultations are an important component of patient care. Informal consultations can occur as spontaneous, face-to-face chats with medical professional colleagues. Several methods to facilitate formal curbside consultation have been developed over the years for psychiatric care [3]. Bergus et al. [4] report on an electronic e-mail service for consultations between primary healthcare physicians and specialists. Carr et al. [5] present details on a consultation liaison service as a way for family physicians to consult with an onsite trainee psychiatrist who provides expert knowledge to family physicians. More recently, alternatives such as telepsychiatry and electronic consultations (eConsults) have been explored. Telepsychiatry uses videoconferencing to conduct psychiatric consultations in real-time (synchronous), or primary care providers (PCP) can record patient information and electronically send to the psychiatrist to review at a later time (asynchronous) [6, 7]. A limitation of synchronous telepsychiatry is that it requires access to high-quality video conferencing software and a strong and consistently reliable internet connection [7] which may not be feasible in remote and rural areas of Canada [2]. Additionally, healthcare professionals spend the same amount of time with each patient as if it were face-to-face, and some patients may not want to be videotaped [7].

eConsults are asynchronous systems which allow for secure web-based communication between PCPs and specialists. As they do not require real-time connections or video processing, they do not necessarily have the same potential bandwidth issues as telepsychiatry. By using eConsult, PCPs can submit questions to specialists, who in turn offer recommendations in a timely manner, thus enabling access with the potential of also avoiding a face-to-face consultation.

Evidence suggests that eConsults are associated with improved access to specialty care and high levels of PCP and patient satisfaction [8, 9]. The Champlain BASE ™ (Building Access to Specialists through eConsultation) eConsult service was developed in Ottawa, Ontario to address excessive wait times, which exceeded 9 months in some specialties for non-urgent cases [10]. The system was launched as a proof of concept in 2010 [11], and was piloted between 2011 to 2012 [12]. The program is now a full service funded through the Ontario Ministry of Health [13]. The Champlain BASE ™ eConsult service has since grown to include 1355 registered PCPs (including 1160 family physicians and 195 nurse practitioners) and 102 specialty services. Over 33,000 eConsults have been completed to date.

The purpose of this study was to analyze all psychiatry eConsults that were completed between April 2011 and January 2015. This paper reports on four specific objectives:

1. To determine the most common clinical topics and types of questions asked;
2. To determine the impact of eConsults on PCP’s course of action and decision to refer;
3. To estimate the number of psychiatry referrals that were avoided through using the eConsult service;
4. To ascertain the perceived value of eConsults by PCPs.

## Methods

### Setting

The Champlain BASE™ eConsult service is a secure online application that facilitates asynchronous communication between PCPs and specialists. The service is based in the Champlain health region of Eastern Ontario, Canada, which has a population of 1.3 million individuals, roughly half of whom reside in the city of Ottawa.

### Champlain BASE™ eConsult service

A PCP creates an eConsult by logging on to the web portal and filling out an electronic form that includes the patient’s medical history and the clinical question being asked to the psychiatrist. PCPs can also attach files, such as laboratory tests, images or videos. A submitted eConsult is then assigned to one of the eConsult psychiatrists based on availability. He or she receives an email alerting them that they have received an eConsult. The specialist then has a week to respond to the PCP within the secure eConsult portal. Specialists can request additional information, provide clinical advice, or advise an in-person consultation. Asynchronous communication can continue between both parties until the PCP decided to close the case within the eConsult service. Following the closing of a case, the PCP is presented with an obligatory close-out survey (Table 1).

**Table 1:** Content of close-out survey for primary care providers

Question	Response Options
Q1. Which of the following best describes the outcome of this eConsultation for your patient:	1. I was able to confirm a course of action that I originally had in mind; 2. I got new advice for a new or additional course of action; 3. I did not find the response very useful; 4. None of the above.
Q2. As a result of the eConsultation would you say that:	1. Referral was originally contemplated but now avoided at this stage; 2. Referral was originally contemplated and is still needed – this eConsult likely leads to a more effective visit; 3. Referral was not originally contemplated and is still not needed – this eConsult provided useful feedback/instruction; 4. Referral was not originally contemplated, but eConsult process resulted in a referral being initiated; 5. There was no particular benefit to using eConsult in this case; 6. Other (please explain).
Q3. Please rate the overall value of the eConsult.	Minimal 1 2 3 4 5 Excellent
Q4. Please rate the overall value of the eConsult service in this case for you as a primary care providers.	Minimal 1 2 3 4 5 Excellent
Q5. We would value any additional feedback you provide:	

Electronic healthcare services, such as the Champlain BASE™ eConsult Service, require that providers are proficient in telespsychiatric competencies [14]. PCPs must be able to present a clear and detailed patient history in order for specialists to confirm and suggest a new management or treatment plan. Over time as specialist advice is implemented and telepsychiatric competencies are applied, entrustment and a working relationship develops [14, 15].

### Study design

This retrospective eConsult review examined all psychiatry eConsults submitted from April 2011 to January 2015. Eligible cases were retrospectively categorized by clinical topic using a hybrid framework of the International Classification for Primary Care, version 2 (ICPC-2) [16] and experiences of the Champlain BASE eConsults medical specialists (see Appendix 1 for our classification taxonomy). The DSM5 was used to classify the various clinical topics that were raised by the PCPs. Ely et al.’s taxonomy of generic clinical questions (TGCQ) [17] was used to classify the “type” of question. The TGCQ uses 4 levels of classification and a total of 64 subcategories to identify the type of question being asked for the first 2 levels of classification and examples of questions. The highest overarching level consists of 6 broad areas, including diagnosis, treatment, management, epidemiology, nonclinical, and unclassified questions. This scale was modified to include the most relevant areas for a referral question. The close-out survey for each eConsult was also included in the dataset. This allowed for the quantification of how the eConsult impacted PCPs course of action and decision for referral, as well as the perceived value of eConsults to PCPs. All data were exported into an Excel spreadsheet and examined through descriptive statistics including frequencies and content analysis for classification of question type.

## Results

Of the 5597 eConsults directed to the service during the study period, 169 were to psychiatry (3.0%). Of these cases, 153 (90.5%) were from family physicians, 150 (88.8%) were from clinicians in urban practices, and 146 (86%) were from female providers. The average response time was just under 3.2 days (median was less than 2.1 days), and the fastest response time was less than 5 min. The majority of eConsults (87.6%) took less than 15 min for the psychiatrist to answer. The vast majority of the eConsults (75%) were completed through a single question/response asynchronous interaction. The remaining consults required more back and forth communication due to the specialist requiring question clarification or more information.

### Clinical topic

Depressive disorders were the most common clinical topic, accounting for 30.2% of all psychiatry eConsults (Table 2). Depressive disorders included all listed in the DSM-5 Depressive Disorder Section: Disruptive Mood Dysregulation Disorder, MDD, Persistent Depressive Disorder, Premenstrual Dysphoric Disorder, Substance/Medication induced Depressive Disorder and Depressive Disorder Due to Another Medical Condition (APA, 2013). Other common clinical topics included Anxiety Disorders (16.6%), Neurodevelopmental Disorders (12.4%), Bipolar and Related Disorders (11.8%), and Schizophrenia Spectrum and Other Psychotic Disorders (10.7%).

**Table 2:** List of content topics in electronic consultation

Content Topic	No. eConsults	% eConsults
Depressive Disorders	51	30.2%
Anxiety Disorders	28	16.6%
Neurodevelopmental Disorders	21	12.4%
Bipolar and Related Disorders	20	11.8%
Schizophrenia Spectrum and Other Psychotic Disorders	18	10.7%
Personality Disorders	5	3.0%
Substance-Related and Addictive Disorders	5	3.0%
Obsessive-Compulsive and Related Disorders	4	2.4%
Disruptive, Impulse-Control, and Conduct Disorders	3	1.8%
Medication-Induced Movement Disorders and Other Adverse Effects of Medication	3	1.8%
Neurocognitive Disorders	3	1.8%
Sleep-Wake Disorders	3	1.8%
Somatic Symptom and Related Disorders	2	1.2%
Feeding and Eating Disorders	1	0.6%
Trauma- and Stressor-Related Disorders	1	0.6%
Unable to classify	1	0.6%

### Question type

Questions pertaining to drug treatment were most common (75.7% of consults). Types of drug treatment questions included drug of choice (39.1% of eConsults), adverse effects from medications (16.0%), how to prescribe a particular drug (5.9%), interactions between medications (3.6%), indications / goals of treating a particular condition (2.4%), and other (8.9%). Questions pertaining to management (13.6%) and diagnosis (10.1%) were less common (Table 3).

**Table 3:** Classification of question type

Question Type	No. eConsults	% eConsults
Drug Treatment		
Drug of choice	66	39.1%
Adverse effects of drugs	27	16.0%
Other	15	8.9%
How to prescribe a particular drug	10	5.9%
Interactions between drugs	6	3.6%
Indications / goals of treating a particular condition	4	2.4%
Drug treatment total	128	75.7%
Clinical Management		
General management question	17	10.1%
Other providers available	6	3.6%
Clinical management total	23	13.6%
Diagnosis		
Interpretation of a clinical finding	11	6.6%
Other	3	1.8%
What test to choose	2	1.2%
Interpretation of a laboratory test	1	0.6%
Diagnosis total	17	10.1%
No specific question	1	0.6%

The questions submitted for the Depressive Disorders – the most common clinical topic– predominately concerned drug treatment (88.2%), particularly *drug of choice* (52.9%). Drug treatment also dominated the other most common clinical areas of Anxiety Disorders (82.1%), Neurodevelopmental Disorders (61.9%), Bipolar and Related Disorders (70%), and Schizophrenia Spectrum and Other Psychotic Disorders (66.7%). *Drug of choice* was the most common drug treatment question across four of these five groups. The exception was the Schizophrenia Spectrum and Other Psychotic Disorders category, where the most common drug treatment questions pertained to *adverse effects of drugs*. Appendix 2 shows de-identified examples of eConsult clinical questions.

### Post-eConsult survey

#### Course of action and decision for referral

PCPs were asked about how the eConsultation affected the course of action for the patient. The majority of the PCPs (62.1%) indicated they received advice for a new or additional course of action, while 32.5% indicated it confirmed a course of action the PCP already had in mind. PCPs were also asked about referrals. In 30.7% of the eConsults, PCPs indicated they no longer needed to refer the patient for a face–to-face appointment based on the information provided by the specialist (Table 4). In 27.8% of eConsults, it was determined that a face-to-face visit was still needed.

**Table 4:** Responses to close-out survey question 2: As a result of the eConsultation would you say that…

Response	No. eConsults	% eConsults
1. Referral was originally contemplated but now avoided at this stage;	52	30.8%
2. Referral was originally contemplated and is still needed – this eConsult likely leads to a more effective visit;	47	27.8%
3. Referral was not originally contemplated and is still not needed – this eConsult provided useful feedback/instruction;	60	35.5%
4. Referral was not originally contemplated, but eConsult process resulted in a referral being initiated;	2	1.2%
5. There was no particular benefit to using eConsult in this case;	5	3.0%
6. Other (please explain).	3	1.8%

#### Overall value

PCPs were asked to rank the overall value in using the eConsultation service for this patient using a Likert scale ranging from 1 (minimal value) to 5 (excellent value). Using this scale, 88.7% of PCPs gave the eConsult service a 4 (27.8%) or a 5 (60.9%) for its value to the patient. One of the reasons PCPs valued the eConsult was that the average response time was much shorter than traditional referrals. A PCP highlighted how one of their patients appreciated the eConsult service:

*“It is great to receive such detailed advice. I was able to have a good discussion with my patient about what the next steps are for her and she is happy to have an early contact with the specialist about her case.”*

Only one PCP reported minimal value to their patient, in the clinical context of patient urgency and safety concerns that required emergency intervention which could not be addressed by the service.

Using a Likert scale of 1 (minimal) to 5 (excellent), PCPs were asked to rate the overall value of the eConsult service in this case to them as a PCP. The majority (91.1%) rated the value to be a 4 (20.7%) or 5 (70.4%). Many PCPs indicated that they found the advice and/or resources provided by specialists to be helpful and efficient. For example:

*“Excellent collaboration, very quick response – so glad to have access to this service!”*

*“Thank you to the specialist for providing effective practical advice and for the research literature information.”*

## Discussion

Our study found that the eConsult psychiatry service is a useful and valued tool to assist in addressing the significant treatment gap for mental illness in primary care settings. In keeping with the evidence for prevalence of mental illness, this study found that depression was the most common clinic topic for which clinical assistance was requested, followed by anxiety, neurodevelopment, bipolar, and schizophrenia spectrum disorders. MDD is the second leading cause of disability worldwide [18], more than breast, colorectal, lung, and prostate cancers [19]. Thus, timely access to care is paramount.

By far the most common questions asked pertained to pharmacotherapy recommendations around how to choose, augment and switch between commonly used psychotropic medications. These included, but were not limited to, frequently prescribed Selective Serotonin Reuptake Inhibitors, Selective Norepinephrine Reuptake Inhibitors, and First and Second Generation Antipsychotics. Questions regarding dosing and side effect profiles were more often asked about Methylphenidate and Amphetamine based Stimulants, Mood Stabilizers and Anticonvulsants. Polypharmacy concerns and medical monitoring were commonly asked across all classes. A possible explanation why there are more treatment type questions than diagnosis questions is that it is harder to easily diagnose patients in psychiatry as specialists do not have investigations to rely upon. Furthermore, PCPs may be able to diagnose cases more easily but need more assistance with treatment options.

In a similar study by Lowenstein et al. [20], the authors describe the implementation of eConsults specifically for psychiatric services. The authors evaluated 50 psychiatry eConsults, which revealed that PCPs most commonly asked questions about depressive disorders (20/50, 40%). (49/50, 98%) were related to the management of the disease, most often for managing medications. The average response time for specialists reported in the Lowenstein study was 1.4 days, slightly lower than the response time reported here, 2.3 days.

In another specialist classification study using eConsult data, Wren et al. [21] found that 46% of PCP cases were related to treatment compared to almost 76% in our analysis. However, the Wren study included nine specialties but no psychiatry questions. Goberstein et al. [22] recently published a study exploring the effects of electronic psychiatric consultations of mental health care. They found that PCPs reported high levels of confidence in treating depression with medication, through referral. However, there is no indication of how many consults were related to treatment.

Analysis of results indicates that the eConsult service had a significant impact on PCPs course of action, as it provided them with advice and/or information that they anticipated would lead to a more effective visit with their patient in a majority of cases. In the study by Lowenstein et al. [20], 38/50 (76%) of PCPs implements the specialists’ recommendation. Although we did not directly ask this question it can be assumed that the vast majority of PCPs implemented the specialist’s advice as it was either new or confirming information (95.3%).This transmission of clinical knowledge may have the potential for capacity building when PCPs encounter similar cases in their practice.

Moreover, if eConsults continue to reduce referrals to specialty services, this has important implications for both patients and the health care system. Benefits could include decreased wait times for patients who require face-to-face specialist care, and cost savings as an alternative model for the delivery of specialty services within the health care system. The service proved to be highly valued by PCPs for its high-quality advice and provision of rapid access to specialist knowledge and recommendations.

Although not the only solution to the treatment gap in the provision of mental health care in the primary care setting, eConsult has demonstrated clinical utility in narrowing the gaps for both patients and providers in Eastern Ontario. Therefore, expanding the service to other healthcare regions in Ontario and across Canada, which our group is currently doing, could yield similar success. Future research analyzing and comparing eConsult analysis between different jurisdictions to identify patterns in the healthcare needs of the population would help to identify gaps in healthcare services.

The findings of this study are susceptible to several limitations. First, as a study completed at a single center in Eastern Ontario, Canada, the result may not be generable to other health urban or rural centers with varying infrastructures. However, the results of the study align with similar studies in other health regions [20]. The data were analyzed retrospectively which also poses as a limitation as some of the consults dated back to 2011. The sample analyzed was a convenience sample, and thus might not have been representative of the population being examined.

## Conclusions

eConsult can facilitate greater collaboration between PCPs and psychiatrists, and as such aligns with recommendations made in the 2011 position paper *The Evolution of Collaborative Mental Health Care in Canada: A Shared Vision for the Future* [23] which was developed by the Canadian Psychiatric Association and the College of Family Physicians of Canada. This paper highlights the importance of effective communication between providers as well as the need for rapid access to advice. It also supports the need to “explore innovative ways for mental health providers to work more closely with PCPs” (p.6). Certainly eConsult represents an excellent example of such an innovation. The Mental Health Commission of Canada similarly recommends the importance of the “medical home” and the need to support collaboration with mental health providers, in recognition of the fact that most mental health care is provided in the primary care setting. eConsult exemplifies a facet of the Canadian collaborative shared care model, which brings together patients, families, primary care providers and mental health providers with the goal of improving mental health outcomes for patients.

## Appendix 1

**Table 5:** Psychiatry content taxonomy for classifying eConsults

Anxiety Disorders	
Bipolar and Related Disorders	
Depressive Disorders	
Dissociative Disorders	
Disruptive, Impulse-Control, and Conduct Disorders	
Elimination Disorders	
Feeding and Eating Disorders	
Gender Dysphoria	
Medication-Induced Movement Disorders and Other Adverse Effects of Medication	
Neurocognitive Disorders	
Neurodevelopmental Disorders	
Obsessive-Compulsive and Related Disorders	
Paraphilic Disorders	
Personality Disorders	
Schizophrenia Spectrum and Other Psychotic Disorders	
Sexual Dysfunctions	
Sleep-Wake Disorders	
Substance-Related and Addictive Disorders	
Trauma-and Stressor-Related Disorders	

## Appendix 2

**Table 6:** Examples of eConsult clinical questions

Depression	
35 year old woman with dysthymia. Has been seeing a psychologist for over a year. Symptoms of low mood, decreased motivation and anhedonia. Not suicidal. No alcohol or drug use. No significant anxiety symptoms. Was started on Prozac and dose increased up to 40 mg without effect. She was then switched to Effexor with dose titrated up to 225 mg with little response. Do you have any suggestions?	
ADHD	
21 year old university student with diagnosis of ADD. Treated with Concerta 54 mg in the morning and Ritalin 30 mg at lunch/early afternoon as he has an evening class. He has asked to increase the dose of Concerta but I am uncomfortable with that as the dose is already high. Otherwise healthy. Takes no other medications. What would you advise?	
Anxiety/Insomnia	
My question is about insomnia. 42 year old woman is severely anxious and reports episodes of panic attacks. She is currently on sertraline 50 mg which is being titrated up but still has primary and secondary insomnia. She has responded to zopiclone 3.25 mg prn for small periods of time but I do not want to continue this due to the risk of tolerance/dependence. She is unable to tolerate trazodone. Is it reasonable to use zopiclone long term? Otherwise would nortriptyline be safe to use with sertraline? If so at what dose? Do you have other suggestions?	

## Abbreviations

Champlain BASE ™: Building Access to Specialists through eConsultation
eConsult: electronic consultations
ICPC-2: International Classification for Primary Care, version 2
MDD: Major Depressive Disorder
PCP: primary care provider
TGCQ: Taxonomy of Generic Clinical Questions
